# Effect of amino groups of mesoporous silica nanoparticles on CpG oligodexynucleotide delivery

**DOI:** 10.1088/1468-6996/16/4/045006

**Published:** 2015-08-18

**Authors:** Yi Xu, Peter Claiden, Yufang Zhu, Hiromi Morita, Nobutaka Hanagata

**Affiliations:** 1 School of Medical Instrument and Food Engineering, University of Shanghai for Science and Technology, 516 Jungong Road, Shanghai 200093, People’s Republic of China; 2 School of Engineering, Sino-British College (USST), 1195 Fuxing Zhong Road, Shanghai 200031, People’s Republic of China; 3 School of Materials Science and Engineering, University of Shanghai for Science and Technology, 516 Jungong Road, Shanghai 200093, People’s Republic of China; 4 Nanotechnology Innovation Station, National Institute for Materials Science, 1-2-1 Sengen, Tsukuba, Ibaraki 305-0047, Japan

**Keywords:** mesoporous silica, amination, CpG delivery, cytokine induction

## Abstract

In this study, we proposed to modify mesoporous silica nanoparticles (MSNs) with 3-aminopropyltriethoxysilane (NH_2_-TES), aminoethylaminopropyltriethoxysilane (2NH_2_-TES) and 3-[2-(2-aminoethylamino)ethylamino] propyl-trimethoxysilane (3NH_2_-TES) for binding of cytosine-phosphate-guanosine oligodexynucleotides (CpG ODN), and investigated the effect of different amino groups of MSNs on the CpG ODN delivery. Serum stability, *in vitro* cytotoxicity, and cytokine interleukin-6 (IL-6) induction by MSN-NH_2_/CpG, MSN-2NH_2_/CpG and MSN-3NH_2_/CpG complexes were investigated in detail. The results showed that three kinds of aminated-MSN-based CpG ODN delivery systems had no cytotoxicity to RAW264.7 cells, and binding of CpG ODN to MSN-NH_2_, MSN-2NH_2_ and MSN-3NH_2_ nanoparticles enhanced the serum stability of CpG ODN due to protection by the nanoparticles. However, three aminated MSN-based CpG ODN delivery systems exhibited different CpG ODN delivery efficiency, and MSN-NH_2_/CpG complexes had the highest ability to induce IL-6 secretion.

## 1. Introduction

Cytosine-phosphodiester-guanine oligodeoxynucleotides (CpG ODN) therapy is a promising therapeutic approach for the treatment of a wide variety of diseases such as cancer, allergy, infectious diseases and arthritis [1, 2]. CpG ODN are short, synthetic and single-stranded DNA sequences containing CpG motifs. CpG ODN can be recognized by Toll-like receptor 9 (TLR9) in antigen-presenting cells (APCs) such as dendritic cells and B cells to activate the innate immune system [3–5]. CpG ODN have been demonstrated to influence several signaling pathways in immune cells, leading to cytokine production in human beings, so they show potential for clinical applications in the treatment of various diseases [6–10]. However, the immunostimulatory effects are often limited by the poor stability of CpG ODN due to easy degradation of CpG OND by nucleases, which limits their clinical application.

Many efforts have been made to enhance the stability of CpG ODN. Chemical modification is one effective technique to protect CpG ODN against degradation by nucleases. For example, the native phosphodiester bond can be replaced with a nuclease-resistant phosphorothioate backbone. However, repeated administration of backbone-modified CpG ODN may cause some side effects, such as reduced immune responses, lymphoid follicle destruction and organ enlargement [11–14]. Recent studies indicated that the poor stability of CpG ODN can be significantly improved using nanobiotechnology. Until now, various nanoparticle-based CpG ODN delivery systems have been reported for delivering CpG ODN to the targets, which can not only protect CpG ODN from degradation and prolong circulation time in the body, but also improve the cellular uptake efficiency of CpG ODN [15–18]. For example, Wei *et al* reported that self-assembled CpG-conjugated Au nanoparticles could deliver CpG ODN into RAW264.7 cells and activate an immune response [15]. Tao *et al* proved that the CpG-Ag complex could be engulfed by RAW264.7 cells and enhanced the induction levels of IL-6 and TNF-*α* [16]. Chen *et al* demonstrated that chitosan-silica nanoparticles could absorb CpG ODN via electrostatic bonding and deliver CpG ODN into 293XL-hTLR9 cells, and thereby activate the higher level of IL-6 production [17].

Mesoporous silica nanoparticles (MSNs), a type of inorganic nanomaterial, are demonstrated to be excellent vehicles for drug/gene delivery [19, 20] due to their high surface area, large pore volume, biocompatibility and their ease of synthesis and surface modification [21–26]. Recent studies showed that the MSN-based delivery system can enhance the delivery efficiency of DNA and protect DNA from degradation [27–32]. For example, Kim *et al* reported that the aminated MSNs can effectively load and deliver plasmid DNA (pDNA) within rat mesenchymal stem cells; the complex showed a high transfection efficiency, and can induce related protein expression [31]. Kar *et al* reported a pDNA delivery system based on polypeptide poly-L-arginine (PLA) modified MSNs. The MSN/PLA complex showed enhanced pDNA delivery efficiency in HeLa and A549 cells, and led to expression of related proteins in the cells [32].

CpG ODN stimulates the innate immune system through different mechanisms; the structure and binding of CpG ODN may affect their immunostimulatory ability. Nishikawa *et al* reported that compared to single-stranded ODN and double-stranded ODN, Y-shaped ODN show a high immunostimulatory effect on RAW264.7 cells, because Y-shaped ODN were more easily taken up by RAW264.7 cells [33]. The Nishikawa group also reported a dendrimer-like DNA. The highly branched structure can effectively interact with TLR9 and further induce a greater amount of TNF-*α* and IL-6 [34]. Chinnathambi *et al* found that cytokine induction was affected by the binding mode of CpG ODN on nanoparticles: CpG ODN bound onto allylamine-modified silicon nanoparticles induced IFN-*α* production, whereas CpG ODN cross-linked to maleimide-modified silicon nanoparticles induced IL-6 [35]. Recently, our group developed a CpG ODN delivery system by binding CpG ODN onto aminopropyl-modified MSNs, which significantly enhanced the level of IL-6 induction [36]. However, studies indicated that different surface modification can influence the delivery efficiency [37, 38]. We suppose that the modification of MSNs with different amino groups could affect the structure or binding of CpG ODN on MSNs, and thereby result in different cytokine induction ability.

**Scheme 1.**
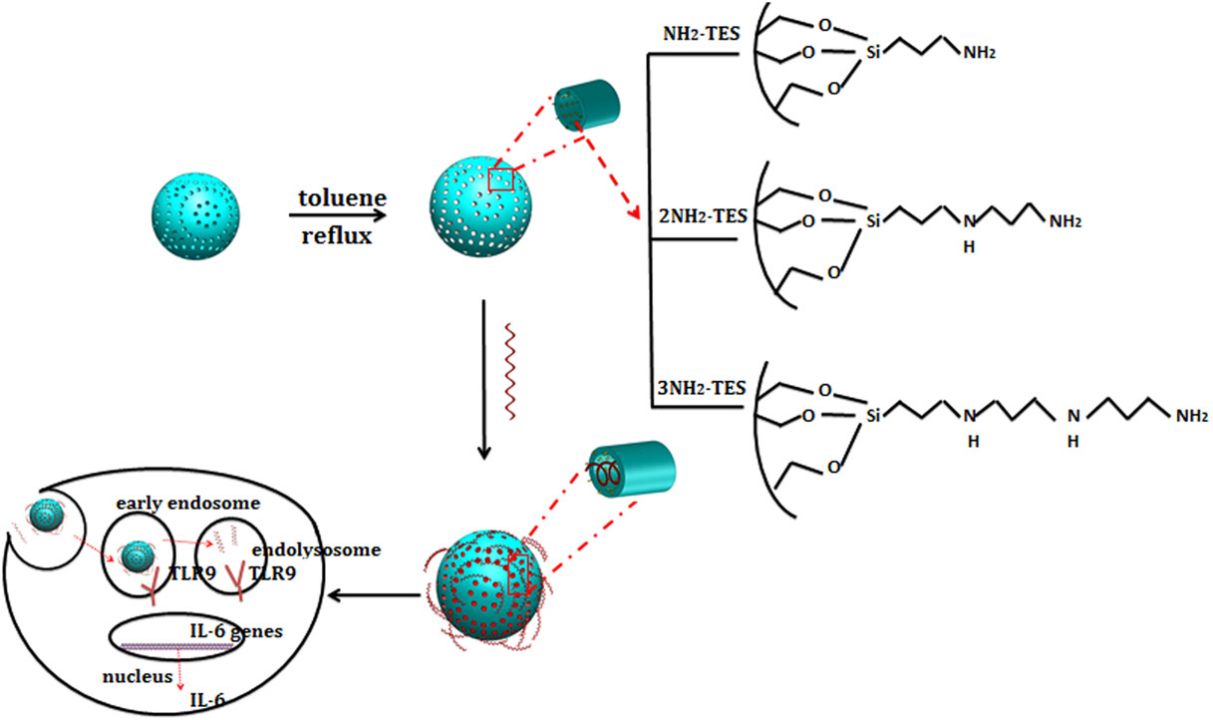
Schematic of aminated MSN-based CpG ODN delivery system.

In this study, we introduced 3-aminopropyltriethoxysilane (NH_2_-TES), aminoethylaminopropyltriethoxysilane (2NH_2_-TES) and 3-[2-(2-aminoethylamino)ethylamino] propyl-trimethoxysilane (3NH_2_-TES) to modify MSNs to form positively charged MSN-NH_2_, MSN-2NH_2_ and MSN-3NH_2_ nanoparticles, and negatively charged CpG ODN was bonded onto these nanoparticles to form MSN-NH_2_/CpG, MSN-2NH_2_/CpG and MSN-3NH_2_/CpG complexes, respectively. RAW264.7 cells were used to culture with three kinds of aminated-MSN-based CpG ODN delivery systems, and *in vitro* cytotoxicity, cellular uptake and the TLR9-mediated induction of IL-6 were investigated (scheme 1). Furthermore, bond dissociation vs time and CpG ODN induction rate of MSN-NH_2_/CpG, MSN-2NH_2_/CpG and MSN-3NH_2_/CpG complexes were analyzed from a theoretical perspective based on Bell’s model.

## 2. Experimental section

### 2.1. Chemicals and materials

Hexadecyltrimethylammonium p-toluenesulfonate (CTAT), 3-aminopropyltriethoxysilane (NH_2_-TES) and 3-[2-(2-aminoethylamino) ethylamino] propyl-trimethoxysilane (3NH_2_-TES) were obtained from Sigma-Aldrich. Tetraethyl orthosilicate (TEOS), triethanolamine (TEA), aminoethylaminopropyltriethoxysilane (2NH_2_-TES), toluene and ethanol were obtained from Sinopharm Chemical Reagent Co. Ltd. Agarose I™, 6×sucrose DNA loading buffer II, 50×TAE buffer, ethidium bromide (EB, 10 mg ml^−1^), fetal bovine serum (FBS), CpG ODN, and ethylenediaminetetraacetic acid (EDTA) disodium salt dihydrate were obtained from Shanghai Sangon Biotech Co. Ltd. Ultrapure water was obtained from a Millipore pure water system. All other chemicals were of analytical reagent grade and were used without further purification.

### 2.2. Synthesis and modification of MSNs

MSNs were prepared according to our recently reported method [36]. Aminated nanoparticles were obtained by modifying MSNs with NH_2_-TES, 2NH_2_-TES and 3NH_2_-TES to form MSN-NH_2_, MSN-2NH_2_ and MSN-3NH_2_ nanoparticles, respectively. A typical experiment for modification of MSNs with NH_2_-TES, 2NH_2_-TES or 3NH_2_-TES was performed as follows: 500 mg of MSNs was suspended in 40 ml of anhydrous toluene by ultrasonication, afterwards the mixture was heated to 100 °C to remove water, and then 3.2 mmol of NH_2_-TES, 2NH_2_-TES or 3NH_2_-TES nanoparticles was added into the mixture. The mixture was refluxed for 20 h under a nitrogen atmosphere. The resulting white solid powder was collected by centrifugation and washed with toluene several times to eliminate un-reacted moieties. Finally, all the as-modified nanoparticles were dried under vacuum at 60 °C for 24 h.

### 2.3. Characterization

Scanning electron microscopy (SEM) was carried out using an FEI Quanta 450 field emission scanning electron microscope. Transmission electron microscopy (TEM) images were obtained on a JEM-2100F microscope. N_2_ adsorption–desorption isotherms were obtained on a Micromeritics Tristar 3020 automated surface area and pore size analyzer at −196 °C under continuous adsorption conditions. Brunauer–Emmett–Teller (BET) and Barrett–Joyner–Halenda (BJH) methods were used to determine the surface area, pore size distribution and pore volume. Dynamic light scattering (DLS) and zeta potential measurements were performed on a Malvern zeta-sizer Nano-ZS90. Fourier transform infrared (FTIR) spectra were recorded on a LAM750(s) spectrometer in transmission mode. UV–vis absorption spectra were measured on a NanoDrop 2000C spectrophotometer. Thermo-gravimetric (TG) analysis was performed on a DMA-8000 dynamic mechanical thermal analyzer at N_2_ atmosphere with a flow rate of 20 ml min^−1^ and a heating rate of 5 C° min^−1^.

### 2.4. Binding of CpG ODN onto MSN-NH_2_, MSN-2NH_2_ and MSN-3NH_2_ nanoparticles (MSN-NH_2_/CpG, MSN-2NH_2_/CpG and MSN-3NH_2_/CpG complexes)

CpG ODN (sequence: 5′-TCAGAGAGTTAGA GAGTTAGAGAGTCAGAGAGTTAGAGA GTTAGAGAGTCAGAGAGTTAGAGAGTTAGAGAG-3′, 72 bases), were diluted in ultrapure water to a concentration of 1 *μ*g *μ*l^−1^ and stored at −20 °C until use. The binding of CpG ODN onto the surface of aminated MSNs was performed at room temperature. Briefly, MSN-NH_2_ nanoparticles were suspended in ultrapure water with a concentration of 1 *μ*g *μ*l^−1^. Subsequently, the as-prepared MSN-NH_2_ suspension dispersed in CpG ODN solution with a fixed weight ratio of MSN-NH_2_/CpG ODN (*R* = 2, 5, 10, 20, 50 or 100), the reaction system was shaken at room temperature for 4 h. Finally, MSN-NH_2_/CpG complexes were collected by centrifugation at 12 000 g for 10 min, and washed with ultrapure water three times to remove the residual free CpG ODN. Binding of CpG ODN onto MSN-2NH_2_ or MSN-3NH_2_ nanoparticles was performed using the same process of MSN-NH_2_ nanoparticles. UV–vis analysis was used to estimate the adsorbed CpG ODN amount by measuring the supernatant of the complexes. The remaining supernatants were also analyzed with gel electrophoresis by loading onto 3% agarose gel with EB and running with loading buffer at 120 V for 10 min.

### 2.5. Stability of MSN-NH_2_/CpG, MSN-2NH_2_/CpG and MSN-3NH_2_/CpG complexes

MSN-NH_2_/CpG, MSN-2NH_2_/CpG and MSN-3NH_2_/CpG complexes were prepared at a CpG ODN concentration of 5 *μ*g *μ*l^−1^ and stored at −20 °C, free CpG ODN with the same concentration were prepared for comparison. The stability experiment was performed as follows. Briefly, 2 *μ*l of free CpG ODN and MSN-NH_2_/CpG, MSN-2NH_2_/CpG, MSN-3NH_2_/CpG complexes containing 10 *μ*g of CpG ODN were incubated in an aqueous solution containing 20% FBS at 37 °C for 0, 2, 5 and 8 h, respectively. After digestion, all samples were subsequently treated with 2 *μ*l of 250 mM EDTA for 2 min at 80 °C to quench the digestion reaction. All samples were collected by centrifugation and washed once with ultrapure water. Finally, the as-prepared product was dispersed in 2 *μ*l of ultrapure water, and then analyzed with gel electrophoresis by loading onto 3% agarose gel with EB and running with loading buffer at 120 V for 10 min.

### 2.6. *In vitro* release behavior of MSN-NH_2_/CpG, MSN-2NH_2_/CpG and MSN-3NH_2_/CpG complexes

*In vitro* release of CpG ODN from MSN-NH_2_/CpG, MSN-2NH_2_/CpG or MSN-3NH_2_/CpG complexes was carried out with a shaking bed at 37 °C. Typically, 180 *μ*g of MSN-NH_2_/CpG, MSN-2NH_2_/CpG or MSN-3NH_2_/CpG complexes was immersed into 180 *μ*l PBS buffer (pH = 7.4) in a 1.5 ml tube, and the tube was then fixed on the shaking bed with a shaking speed of 150 rpm. After a predetermined time interval, 3 *μ*l of the suspension was collected and centrifuged, and the supernatant was used for quantitative analysis of the released CpG ODN with a NanoDrop 2000C spectrometer.

### 2.7. Cell culture

RAW264.7 cells were purchased from InvivoGen (San Diego, CA, USA), and grown in Dulbecco’s modified Eagle’s medium (DMEM, Sigma-Aldrich) supplemented with 10% FBS, 50 U ml^−1^ penicillin, 50 mg l^−1^ streptomycin, 100 *μ*g ml^−1^ normocin and 10 *μ*g ml^−1^ blasticidin at 37 °C in humidified air containing 5% CO_2_. RAW264.7 cells were cultured according to the manufacturer’s instructions.

### 2.8. *In vitro* cytotoxicity assay

An *in vitro* cytotoxicity assay for MSN-NH_2_, MSN-2NH_2_, and MSN-3NH_2_ nanoparticles was performed using a Cell Counting Kit-8 (CCK-8, Dojindo, Japan). RAW264.7 cells were seeded into a 96-well plate at a density of 3.3 × 10^4^ cells per well. After seeding the cells, the MSN-NH_2_, MSN-2NH_2_ and MSN-3NH_2_ nanoparticle solution (1 mg ml^−1^ in DMEM) was immediately added into a 96-well plate. The final concentrations of MSN-NH_2_, MSN-2NH_2_ and MSN-3NH_2_ nanoparticles were 0, 25, 50, 75 and 100 *μ*g ml^−1^, and the final medium volume in each well was 100 *μ*l. After incubation of cells for 24 h, 10 *μ*l of CCK-8 solution was added into each well, and the cells were incubated for another 2 h. The absorbance at 450 nm was then measured using a microplate reader (MTP-880 Lab, Corona, Japan). Cytotoxicity was expressed as the percentage of viable cells compared with that of untreated control cells.

### 2.9. Cytokine assay

RAW264.7 cells were seeded into a 96-well plate at a density of 1 × 10^5^ cells per well in DMEM. After incubation of cells for 24 h, the cells were stimulated with MSN-NH_2_/CpG, MSN-2NH_2_/CpG and MSN-3NH_2_/CpG complexes (CpG ODN concentration: 2.5 *μ*g ml^−1^). For control, free CpG ODN were added in the culture medium at equal concentration of CpG ODN. After 24 h of incubation at 37 °C, the cells were washed with PBS twice, and total RNA was extracted using Isogen solution (Wako, Japan) and treated with a DNase I digestion step according to the manufacturer’s instructions. The obtained RNA was reverse transcribed into complementary DNA (cDNA) using a primeScript^TM^ RT reagent Kit (Takara, Japan). The cDNA was analyzed for marker of IL-6. Glyceraldehyde3-phosphate dehydrogenase (Gapdh) was utilized as a reference. RT-PCR was performed using a LightCyclerFastStart DNA MasterSYBER Green I Kit (Roche Appl. Sci. Japan). The relative expression level for IL-6 was normalized against the initial concentration value of the reference gene Graph and determined using the second derivative maximum method.

### 2.10. Estimation of data-fit based on a theoretical perspective

Binding energy of MSN-NH_2_/CpG, MSN-2NH_2_/CpG and MSN-3NH_2_/CpG complexes and probability of CpG ODN dissociation from aminated MSNs were analyzed from a theoretical perspective based on Bell’s model [39]. In order to extend Bell’s model to IL-6 induction by CpG ODN in the cells, two conceptual models were proposed. In one, the ends of the aminated molecules on the MSN surface are electrostatically bound to the phosphate groups on the CpG ODN by multiple parallel bonds. It is further assumed that the number of bonds depends on the number of n[NH_2_] groups in the aminated molecule (*n* = 1, 2 or 3). In another model, it is assumed that transfer of CpG ODN to the TLR9 receptor molecule [35] starts with dissociation of the NH_2_ binding sites and that Bell’s model can estimate the probability and rate of dissociation.

## 3. Results and discussion

### 3.1. Synthesis and modification of MSNs

**Figure 1.**
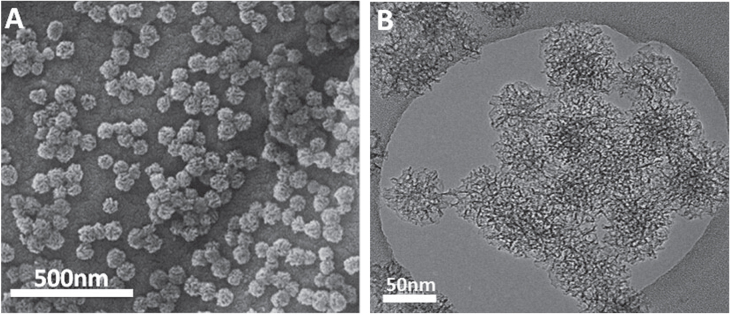
(A) SEM and (B) TEM images of mesoporous silica nanoparticles (MSNs).

The representative SEM and TEM images of MSNs are shown in figure 1. Similar to the previous reported results [36], the average particle size of the spherical MSNs was in the range of 60–70 nm, and mesoporous structure with center-radial pore channels can be observed on MSNs, which facilitates CpG molecules to diffuse into channels, and thereby increases CpG loading. MSNs were then modified with NH_2_-TES, 2NH_2_-TES and 3NH_2_-TES to form MSN-NH_2_, MSN-2NH_2_ and MSN-3NH_2_ nanoparticles. As shown in N_2_ physisorption measurements (figure S1), MSNs and aminated MSNs showed the characteristic type IV isotherms, indicative of a mesoporous structure. The BET surface area of MSNs is 805 m^2^ g^−1^, while the BET surface areas of MSN-NH_2_, MSN-2NH_2_ and MSN-3NH_2_ nanoparticles decreased to be 220, 279 and 316 m^2^ g^−1^, respectively, due to the modification of amino groups on the surface of MSNs (table 1). Compared with the aminated MSNs by a reaction between MSNs and silane in ethanol [36], the surface areas of the MSN-NH_2_, MSN-2NH_2_ and MSN-3NH_2_ nanoparticles are slightly lower, which may be due to the higher number of amino groups grafted onto the MSNs. However, MSN-NH_2_, MSN-2NH_2_ and MSN-3NH_2_ nanoparticles had large mesopore size distributions (>30 nm) except small mesopores (about 3 nm), making it possible to load a large amount of CpG ODN.

**Table 1.** The structural parameters of the MSNs and MSN-NH_2_, MSN-2NH_2_ and MSN-3NH_2_ nanoparticles.

Sample	*S*_BET_ (m^2^ g^−1^)	*V*_P_ (cm^3^ g^−1^)	*D*_P_ (nm)
MSN	805	2.5	3/35
MSN-NH_2_	220	1.06	3/35
MSN-2NH_2_	279	1.15	3/33
MSN-3NH_2_	316	1.17	3/32

**Figure 2.**
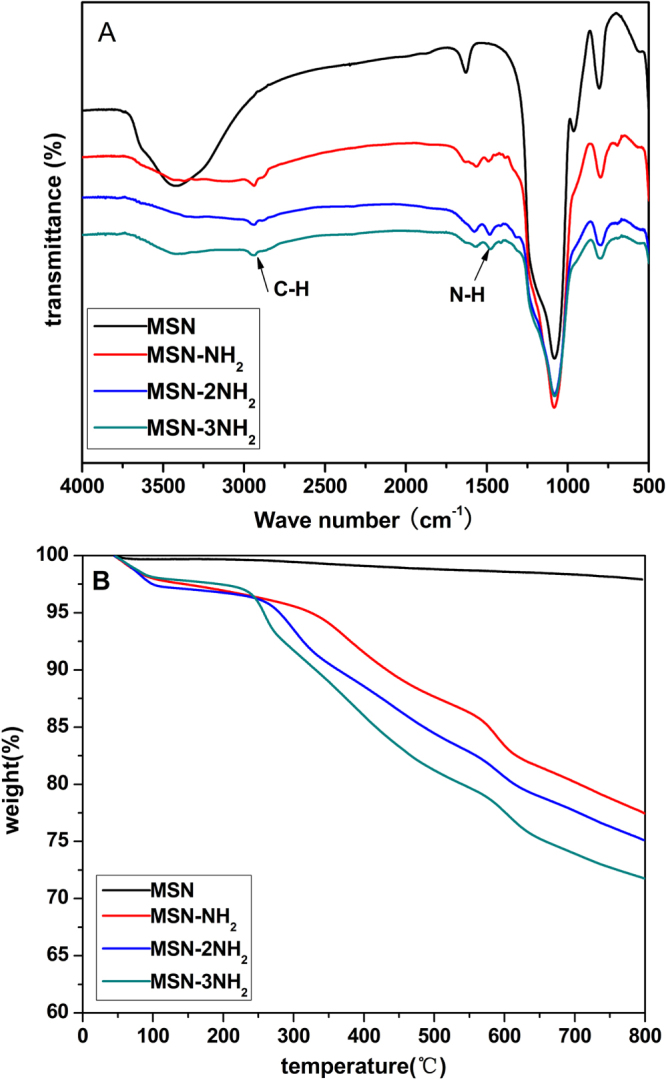
(A) FTIR spectra and (B) TG analysis of MSN, MSN-NH_2_, MSN-2NH_2_ and MSN-3NH_2_ nanoparticles.

FTIR spectra and TG analysis further confirmed the successful surface modification with NH_2_-TES, 2NH_2_-TES and 3NH_2_-TES (figure 2). From the FTIR spectra (figure 2(A)), the Si–OH band at 960 cm^−1^ present in MSNs almost disappears after the modification with NH_2_-TES, 2NH_2_-TES and 3NH_2_-TES, and a vibration peak at 1560 cm^−1^ can be observed on the spectra of MSN-NH_2_, MSN-2NH_2_ and MSN-3NH_2_ nanoparticles, which is assigned to N–H bending vibrations of the amine functional groups. TG analysis (figure 2(B)) showed that the MSNs showed a 2% weight loss from room temperature to 800 °C, whereas that of the MSN-NH_2_, MSN-2NH_2_ and MSN-3NH_2_ nanoparticles showed a 23%, 25% and 28% weight loss, respectively, in the same temperature range, suggesting the successful grafting of amino groups onto the MSNs. Furthermore, the weight loss of MSN-NH_2_, MSN-2NH_2_ and MSN-3NH_2_ nanoparticles increased progressively, because the organic chain became longer as the number of NH_2_ groups increased. According to the weight loss and molecular weight of the organic chain of NH_2_-TES, 2NH_2_-TES and 3NH_2_-TES, it can be estimated that the amounts of grafted NH_2_, 2NH_2_ and 3NH_2_ functional groups were very close to each other.

Zeta potentials were measured when all samples were dissolved in ultrapure water with a concentration of 1 *μ*g *μ*l^−1^. As shown in table 2, the zeta potential of MSNs was −19.5 mV, while those of the MSN-NH_2_, MSN-2NH_2_ and MSN-3NH_2_ nanoparticles were 9.6 mV, 12.3 mV and 15.9 mV, respectively; suggesting the positively charged amino groups have been grafted on the surface of MSNs. Therefore, the positively charged particles could be able to interact with the negatively charged CpG ODN. On the other hand, dynamic light scatting (DLS) analysis revealed that after modified with amino groups, MSNs induced a slight increase in the dynamic particle size, and the polydispersity index (PDI) of aminated MSNs (ranging from 0.2–0.39) shows a slight increase than MSNs (0.17), suggesting that amino modification did not induce more aggregates of aminated MSNs (table 3).

**Table 2.** Zeta potentials of the MSNs and aminated MSNs before and after amino modification and CpG ODN binding (*R* = 2).

Samples	Ave zeta potential (mV)
MSN	−19.5
MSN-NH_2_	9.6
MSN-2NH_2_	12.3
MSN-3NH_2_	15.9
MSN-NH_2_/CpG	−9.2
MSN-2NH_2_/CpG	−10.7
MSN-3NH_2_/CpG	−13.5

**Table 3.** Particle size of aminated MSNs before and after CpG ODN binding (*R* = 2).

Sample name	Ave particle size (nm)	PDI
MSN	89	0.17
MSN-NH_2_	122	0.39
MSN-2NH_2_	129	0.34
MSN-3NH_2_	154	0.20
MSN-NH_2_/CpG	134	0.04
MSN-2NH_2_/CpG	151	0.43
MSN-3NH_2_/CpG	178	0.29

### 3.2. Binding and serum stability of MSN-NH_2_/CpG, MSN-2NH_2_/CpG and MSN-3NH_2_/CpG complexes

**Figure 3.**
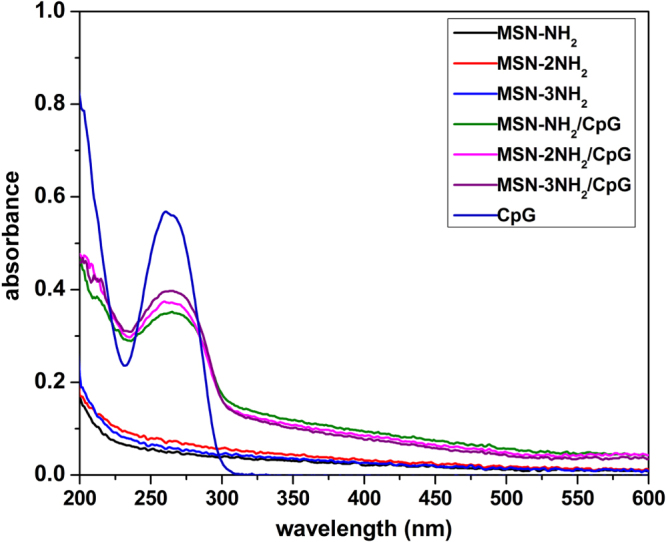
UV–vis spectra of free CpG ODN and aminated MSNs before and after CpG ODN binding (*R* = 6).

Binding CpG ODN onto nanoparticles is important to improve the poor stability of free CpG ODN. Various nanoparticles have been developed as carriers for CpG ODN delivery. Among these carriers, MSNs with large pore size can not only significantly protect CpG ODN from DNase I degradation but can deliver a large amount of CpG ODN to the target. In this study, UV–vis analysis, zeta potential analysis and gel electrophoresis were used to characterize the binding of CpG ODN on aminated MSNs. Figure 3 shows the UV–vis adsorption spectra of MSN-NH_2_, MSN-2NH_2_ and MSN-3NH_2_ nanoparticles before and after CpG ODN binding. It can be observed that MSN-NH_2_/CpG, MSN-2NH_2_/CpG and MSN-3NH_2_/CpG complexes show the characteristic UV–vis absorption peak of CpG ODN at 260 nm, suggesting the CpG ODN was successfully bound to the aminated MSNs. The zeta potential of nanoparticles after CpG ODN binding caused a reversal change, which also confirms the successful absorption of CpG ODN on the aminated MSNs.

**Table 4.** Maximum probability density, dissociation time at maximum probability for bond dissociation of MSN-NH_2_/CpG, MSN-2NH_2_/CpG and MSN-3NH_2_/CpG complexes.

Sample name	Maximum probability density of dissociation	Dissociation time at maximum probability (s)
MSN-NH_2_/CpG	4.5	0
MSN-2NH_2_/CpG	1.5	0.24
MSN-3NH_2_/CpG	1.15	0.48

**Figure 4.**
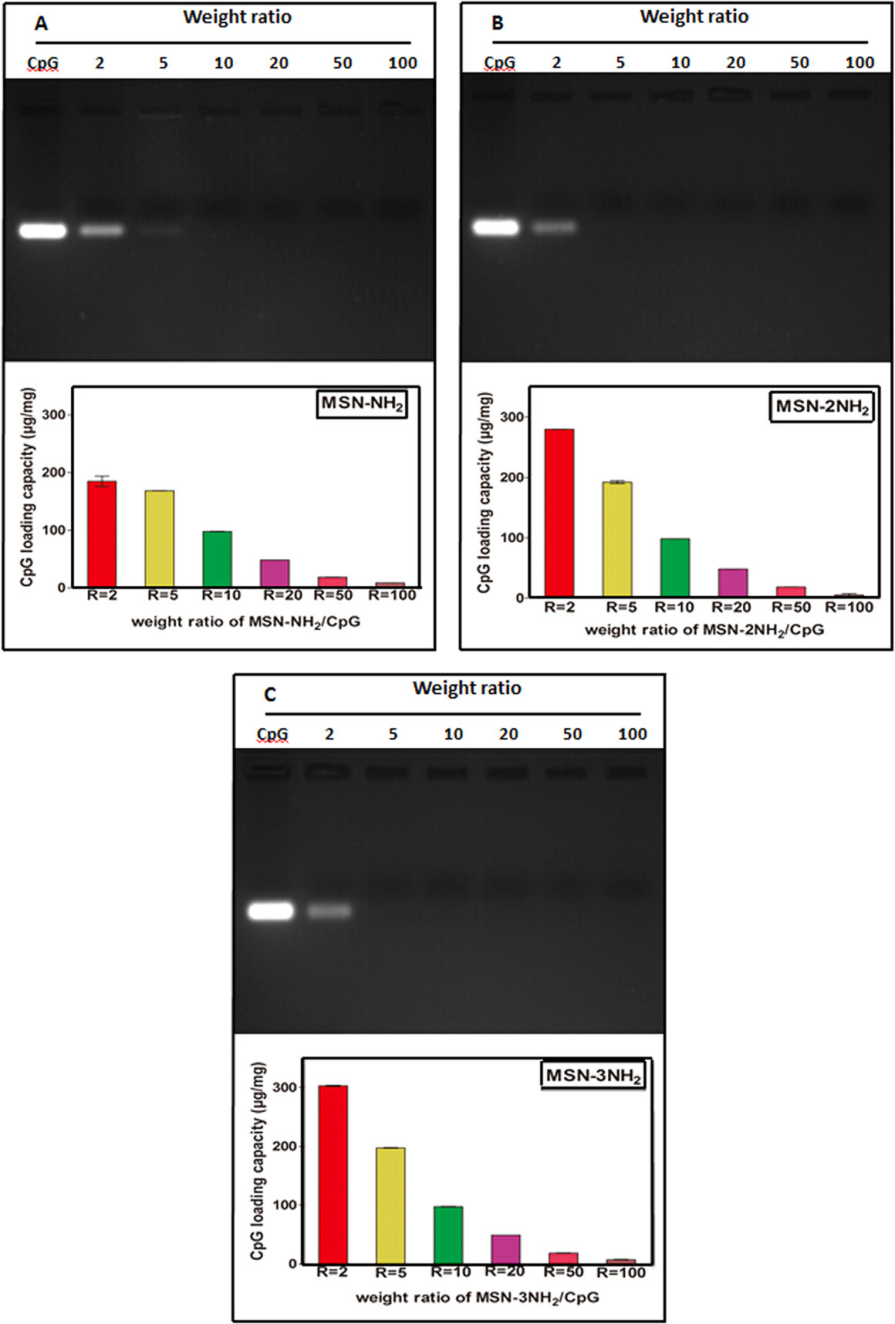
(Upper panels) Agarose gel electrophoresis of the supernatants after binding CpG ODN onto MSN-NH_2_, MSN-2NH_2_ and MSN-3NH_2_ nanoparticles at various weight ratios: (A) MSN-NH_2_/CpG, (B) MSN-2NH_2_/CpG and (C) MSN-3NH_2_/CpG. (Lower panels) The corresponding CpG ODN loading capacities on MSN-NH_2_, MSN-2NH_2_ and MSN-3NH_2_ nanoparticles at various weight ratios: (A) MSN-NH_2_/CpG, (B) MSN-2NH_2_/CpG and (C) MSN-3NH_2_/CpG.

On the other hand, agarose gel electrophoresis of the supernatant after binding CpG ODN onto aminated MSNs are shown in figure 4. The CpG ODN band in the supernatant of MSN-NH_2_ nanoparticles disappeared at a weight ratio of ≥10, while the bands in the supernatants of MSN-2NH_2_ and MSN-3NH_2_ nanoparticles disappeared at a weight ratio of ≥5, which indicates that CpG ODN were able to bind onto the aminated MSNs, but the loading capacities were different. Thus, the loading capacities of CpG ODN on the aminated MSNs were determined by UV–vis analysis (figure 4). The saturation loading capacity of CpG OND on MSN-NH_2_ nanoparticles at a weight ratio of 5 was estimated to be about 168.7 ± 0.4 *μ*g mg^−1^ (figure 4(A)), and the saturation loading capacities of MSN-2NH_2_ and MSN-3NH_2_ nanoparticles at a weight ratio of 2, which is slightly higher than MSN-NH_2_ nanoparticles, were estimated to be about 279.8 ± 0.2 *μ*g mg^−1^ and 303.1 ± 1.6 *μ*g mg^−1^, respectively (figures 4(B) and (C)). The high loading capacities of all three kinds of aminated MSNs might be attributed to the large mesoporous pores, and perhaps the higher the zeta potential of the aminated MSNs, the larger the amount of CpG ODN loading capacity.

**Figure 5.**
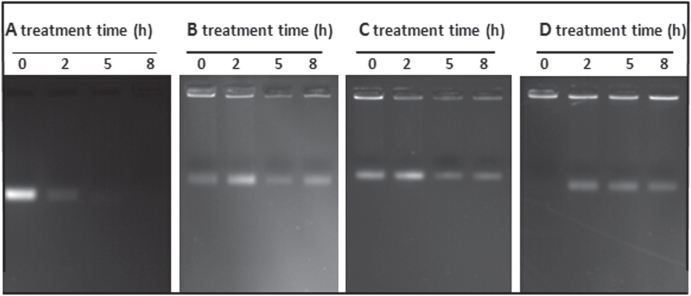
Serum stability of (A) free CpG ODN, (B) MSN-NH_2_/CpG, (C) MSN-2NH_2_/CpG and (D) MSN-3NH_2_/CpG (*R* = 6) in 20% serum-containing media, as measured by agarose gel electrophoresis.

The poor stability of free CpG ODN limits the immunostimulatory effect and is the major drawback restricting their clinical application [40]. Studies demonstrated that nanoparticles, such as gold nanoparticles [15, 41, 42], silver nanoparticles [16], gelatin nanoparticles [43] and silica nanoparticles [28, 38], can be used to deliver genes and proteins to targets. Therefore, binding of immunostimulatory CpG ODN onto aminated MSNs will surely enhance the CpG ODN stability. Serum stability of free CpG ODN, and MSN-NH_2_/CpG, MSN-2NH_2_/CpG and MSN-3NH_2_/CpG complexes were tested in 20% serum-containing medium using gel electrophoresis. As shown in figure 5(A), the free CpG ODN band in the supernatant became weaker with increasing treatment time, and completely disappeared after 5 h digestion. However, CpG ODN bands of MSN-NH_2_/CpG, MSN-2NH_2_/CpG and MSN-3NH_2_/CpG complexes can be clearly observed in each gel well and channel even after 8 h digestion, which indicated that the aminated MSN-based CpG ODN delivery system was able to protect CpG ODN against degradation by nuclease (figures 5(B), (C) and (D)). Herein, the CpG ODN bands of MSN-NH_2_/CpG, MSN-2NH_2_/CpG and MSN-3NH_2_/CpG complexes were located in each gel channel except inside each gel well, which might be attributed to the detached CpG OND from the aminated MSNs. The electrostatic interactions applied to CpG ODN absorbed on the outer surface of aminated MSNs are not strong enough to keep all CpG ODN adsorbed onto the aminated MSNs under the applied electrical field.

### 3.3. *In vitro* cytotoxicity, and IL-6 induction of MSN-NH_2_/CpG, MSN-2NH_2_/CpG and MSN-3NH_2_/CpG complexes

**Figure 6.**
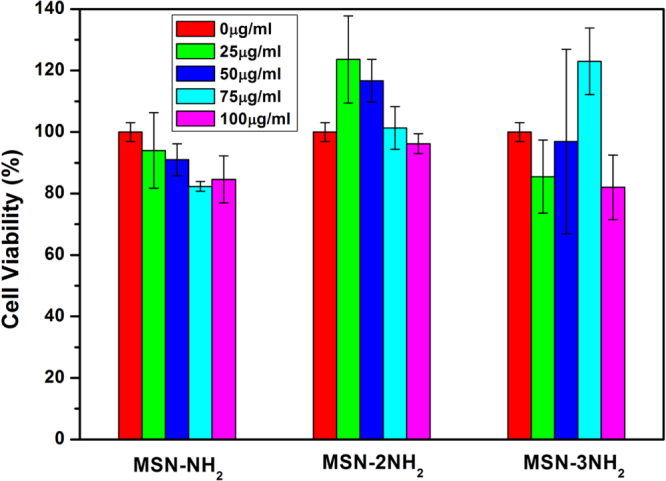
Effect of different concentrations of MSN-NH_2_, MSN-2NH_2_ and MSN-3NH_2_ nanoparticles on the cytotoxicity to RAW264.7 cells, as measured by a Cell Counting Kit-8 assay.

Investigation of the biological safety of drug delivery vehicles is critical for drug delivery. In this study, the potential cytotoxicity of MSN-NH_2_, MSN-2NH_2_ and MSN-3NH_2_ nanoparticles to RAW264.7 cells was evaluated using a Cell Counting Kit-8 (CCK-8) assay. As shown in figure 6, no detrimental effects of MSN-NH_2_, MSN-2NH_2_ and MSN-3NH_2_ nanoparticles on RAW264.7 cells were observed even at a concentration of 100 *μ*g ml^−1^ after incubation up to 24 h, which suggests that MSN-NH_2_, MSN-2NH_2_ and MSN-3NH_2_ nanoparticles are safe and could be used as a non-viral carrier for CpG ODN delivery.

**Figure 7.**
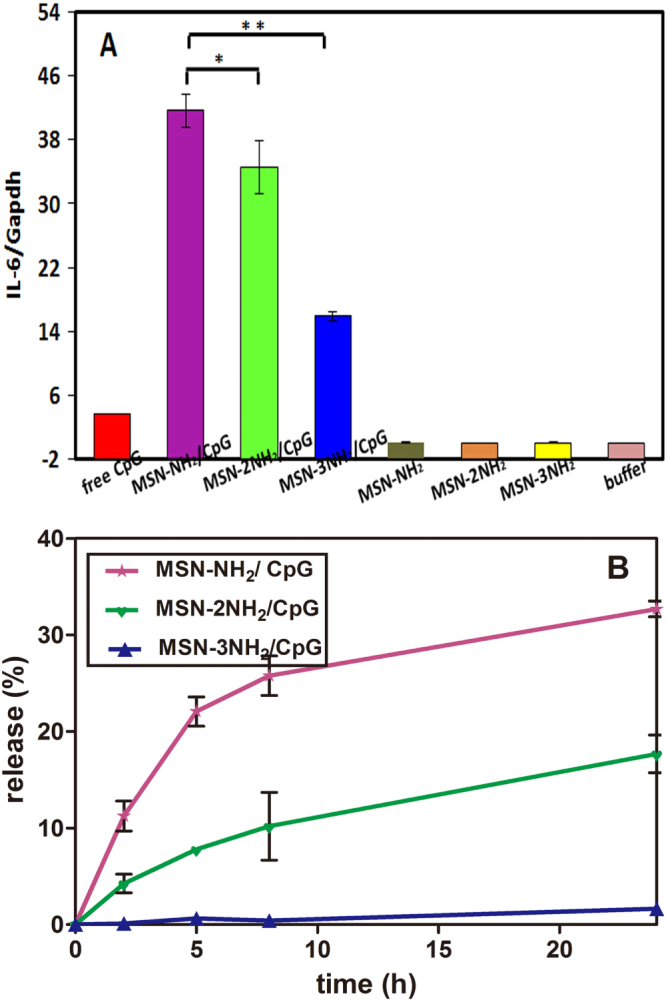
(A) IL-6 induction by RAW264.7 cells cultured with free CpG ODN, MSN-NH_2_/CpG, MSN-2NH_2_/CpG and MSN-3NH_2_/CpG complexes (*R* = 6). (B) The release percentages of CpG ODN from MSN-NH_2_/CpG, MSN-2NH_2_/CpG and MSN-3NH_2_/CpG complexes (*R* = 6), ^∗^*P* < 0.05, ^∗∗^*P* < 0.01.

Subsequently, we tested the TLR9-mediated IL-6 induction abilities of MSN-NH_2_/CpG, MSN-2NH_2_/CpG and MSN-3NH_2_/CpG complexes, which were evaluated using MSN-NH_2_/CpG, MSN-2NH_2_/CpG and MSN-3NH_2_/CpG complexes with a CpG ODN concentration of 2.5 *μ*g ml^−1^ in culture medium for 24 h to stimulate RAW264.7 cells. The bonded weight ratio of aminated MSNs to CpG ODN was taken to be 6, which means that the CpG ODN can be totally absorbed onto the aminated MSNs, and the CpG ODN loading amounts were very close to each other. As a control, the cells were also stimulated by free CpG ODN with the same amount of CpG ODN, the MSN-NH_2_, MSN-2NH_2_ and MSN-3NH_2_ nanoparticles and buffer. As shown in figure 7(A), the buffer and the MSN-NH_2_, MSN-2NH_2_ and MSN-3NH_2_ nanoparticles did not stimulate IL-6 induction, suggesting that the culture medium and carriers do not stimulate cytokine induction, and free CpG ODN induced a low level of IL-6 induction due to the poor stability of free CpG ODN in the culture medium. However, MSN-NH_2_/CpG complexes exhibited an excellent ability to stimulate IL-6 induction (41.7 ± 2.1), which is significantly higher than MSN-3NH_2_/CpG (15.9 ± 0.6). This may due to the different binding modes of CpG ODN on the three kinds of aminated MSNs. Chinnathambi *et al* reported that the binding mode of CpG ODN can significantly affect the induction of cytokines, and IL-6 is more likely to be induced by free CpG ODN [35]. Figure 7(B) shows CpG ODN release from MSN-NH_2_/CpG, MSN-2NH_2_/CpG and MSN-3NH_2_/CpG complexes. It can be found that compared to MSN-3NH_2_ nanoparticles, CpG ODN bonded onto MSN-NH_2_ nanoparticles quickly released in the first 10 h, and then the release became much slower. On the other hand, the release of CpG ODN from MSN-3NH_2_ nanoparticles was slow because the electrostatic forces between CpG ODN and MSN-3NH_2_ nanoparticles were too strong. As mentioned above, IL-6 is more likely to be induced by the released CpG ODN, so the MSN-NH_2_/CpG complexes had better a ability to induce IL-6 secretion.

### 3.4. Binding energy and cytokine induction of MSN-NH_2_/CpG, MSN-2NH_2_/CpG and MSN-3NH_2_/CpG complexes based on a theoretical perspective

**Figure 8.**
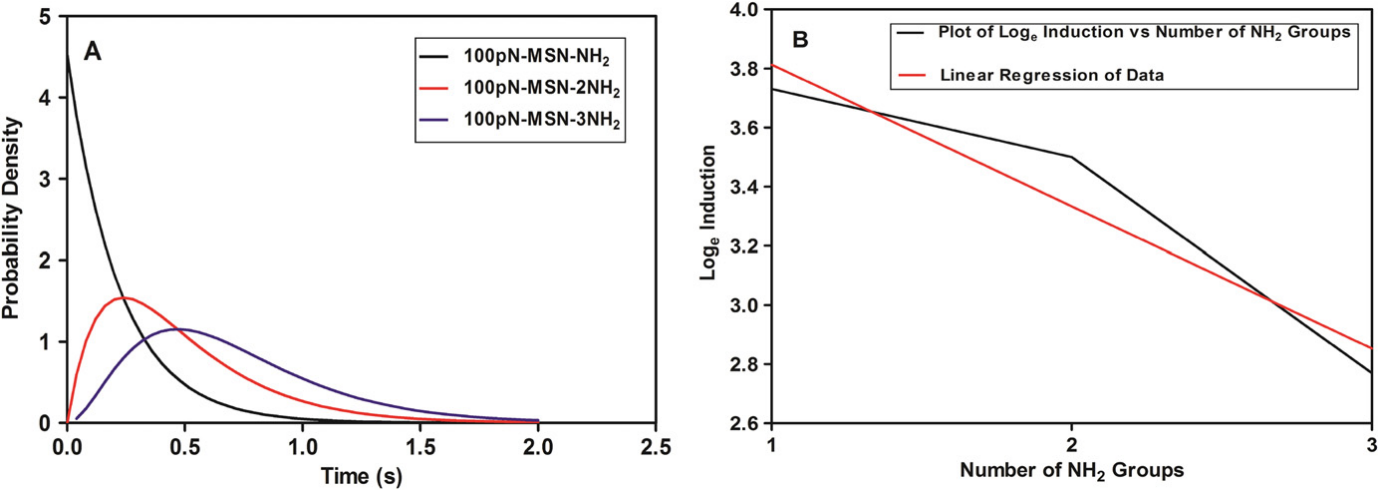
(A) Probability density of bond dissociation vs time for MSN-NH_2_/CpG, MSN-2NH_2_/CpG, and MSN-3NH_2_/CpG complexes. (B) CpG ODN induction rate (log_e_) of MSN-NH_2_/CpG, MSN-2NH_2_/CpG, and MSN-3NH_2_/CpG complexes. Linear regression provides an approximate exponential data-fit of 45.28 exp (−0.48(*x* − 11)) where *x* is the number in the NH_2_ groups 1, 2 or 3.

Induction rates for CpG ODN from functionalized MSNs decreases with increasing complexity of the amino group [44] because the dissociation of CpG ODN occurs at the amino/phosphate electrostatic bond; the strength of this bond depends on the number of amino groups on the functional molecule. Figure 8(A) shows the probability density of dissociation for 1, 2 and 3 binding sites representing MSN-NH_2_, MSN-2NH_2_ and MSN-3NH_2_ nanoparticles respectively [39]. The result shows decreasing (maximum) probability of dissociation with number of amino groups in the aminated MSNs. This is reflected in the decrease of induction rates for IL-6 with number of the NH_2_ groups as shown in figure 7(A), MSN-3NH_2_ nanoparticles exhibit the highest strength and CpG ODN bonded onto MSN-3NH_2_ nanoparticles are less likely to dissociate, and as a result the release percentage of CpG ODN from aminated MSNs is similar to the theoretical perspective (figure 7(B)). While these concurrent trends do not provide a deterministic link between induction and number of NH_2_ groups, they do support the concept that an increased number of binding sites, hence binding energy, will reduce the probability of dissociation and consequently fewer separations of CpG ODN from the MSNs to the TLR9 receptor molecule. The data in figure 8(A) were based on a dissociative force of 100 pN. The actual force will depend on the geometries of the CpG ODN and transporter molecules, and their relative dipole moments. A lower dissociative force would show a similar decrease in probability of bond dissociation with increased number of NH_2_ groups but with less variation between them, and all groups would have a lower probability of dissociation. Under the assumed force, probability densities for dissociation and time at the maximum probability of break-up were analysed.

Figure 8(B) shows the relationship between CpG ODN induction rate (log_e_) and number of amino groups on the particles using the data in figure 7(A). Based on a linear regression of this log–linear model of the data, a formula of 45.28 exp (−0.48(*x* − 1)) provides an approximate fit to induction rate, where *x* is 1, 2 or 3 equivalent to MSN-NH_2_, MSN-2NH_2_ and MSN-3NH_2_ in the aminated functional molecule. This has an exponential form, and it is proposed that an exponential fit to the data is preferred over a general power law fit. Thus, CpG ODN induction rate by MSN-NH_2_ nanoparticles was estimated to be about 45.28, while the rate decreases to 28 by MSN-2NH_2_ nanoparticles and 17 by MSN-3NH_2_ nanoparticles; this indicates that MSN-NH_2_ nanoparticles exhibit a better ability to induce IL-6.

## 4. Conclusions

In this study, we modified MSNs with NH_2_-TES, 2NH_2_-TES and 3NH_2_-TES for CpG ODN binding to form MSN-NH_2_/CpG, MSN-2NH_2_/CpG, MSN-3NH_2_/CpG complexes, and investigated the effect of different amino groups of MSNs on the CpG ODN delivery efficiency. All three types of aminated-MSN-based CpG ODN delivery systems had a high CpG ODN loading capacity, and the serum stability of CpG ODN was significantly enhanced due to the protection by aminated MSNs. An *in vitro* cytotoxicity assay showed that all aminated MSNs had no cytotoxicity to RAW264.7 cells. Most importantly, different amino groups on MSNs could affect the CpG ODN delivery efficiency, and MSN-NH_2_/CpG complexes exhibited the best ability to stimulate IL-6 induction.
